# Research on the impact of the promotion of ideological and political education in university mathematics courses and the stimulation of learning interest on learning effectiveness

**DOI:** 10.3389/fpsyg.2025.1545198

**Published:** 2025-05-16

**Authors:** Liu Cheng, Ji Zhenyi

**Affiliations:** Department of Basic Teaching, Sichuan Agricultural University, Chengdu, China

**Keywords:** university mathematics, learning interest, ideological and political education in courses, self-efficacy, learning persistence, learning anxiety, learning effectiveness

## Abstract

The learning effectiveness of college mathematics is influenced by multiple factors. Starting from the perspective of stimulating learning interest and promoting curriculum-based ideological and political education, a structural equation model was used to study the impact of learning interest and curriculum-based ideological and political education on the learning effectiveness of mathematics. The research results show that (1) both learning interest (standardized direct effect *β* = 0.512, *p* < 0.001) and curriculum-based ideological and political education (standardized direct effect *β* = 0.127, *p* < 0.001) have a significant positive impact on the learning effectiveness of mathematics. In terms of the total effect, the total effect of learning interest on learning effectiveness is 0.581, and the total effect of curriculum-based ideological and political education on learning effectiveness is 0.240. It can be seen that learning interest has a more significant impact on learning effectiveness; (2) Self-efficacy (standardized direct effect *β* = 0.460, *p* < 0.001) and learning persistence (standardized direct effect *β* = 0.495, *p* < 0.001) both have a significant positive impact on the learning effectiveness of mathematics, while learning anxiety (standardized direct effect *β* = 0.404, *p* < 0.001) has a significant negative impact on the learning effectiveness of mathematics; (3) Self-efficacy, learning persistence, and learning anxiety all fully mediate the relationship between learning interest and learning effectiveness, as well as the relationship between curriculum-based ideological and political education and learning effectiveness. The corresponding paths of each mediating effect were tested by the Bootstrap asymmetric confidence interval significance test with 2000 sampling times. The confidence intervals do not contain 0, indicating that the mediating effects are significant. In college mathematical teaching activities, it is necessary to effectively carry out curriculum-based ideological and political education, arouse students’ interest in mathematics learning, cultivate students’ good sense of mathematical self-efficacy, learning persistence, and self-regulation ability, and continuously improve the learning effectiveness of college mathematics.

## 1 Problem proposition

In mathematical learning activities, mathematical learning interest, self-efficacy, learning persistence, and learning anxiety have obvious impacts on learning effectiveness, manifested as the influence of internal motivation and reflecting the internal pulling effect, while ideological and political education in courses, in addition to embodying the purpose of ideological and political education, shows an external pushing effect on mathematical self-efficacy, learning anxiety, learning persistence, and learning effectiveness.

Learning interest is manifested as learning initiative and is a psychological reaction of internal, non-intellectual factors in students’ learning activities (Chunxing, 2003; Guangming et al., 2015; Xuming, 2011). Mathematical learning interest is the “psychological tendency” in students’ mathematical learning process. At the university stage, since students’ minds are more mature, if students have high learning interest, they will have higher attention, effort, and learning participation in the learning process. Research shows that there are significant influence relationships between mathematical learning interest and mathematical self-efficacy and mathematical learning effectiveness (Shengqing and Chunxia, 2018; Peng and Lihua, 2021). Thus, in mathematical teaching activities, how teachers effectively stimulate students’ learning interest is an important part of the reform of university mathematics teaching. Currently, the relationship between college students’ mathematical learning interest and self-efficacy, learning persistence, and learning effectiveness is still worthy of further in-depth exploration.

Self-efficacy stems from social cognitive theory and has an impact on the cognition, motivation, and emotion of social individuals (Bandura, 2022). Mathematical self-efficacy is the subjective judgment and evaluation of mathematical learners on whether they can complete mathematical learning, control learning behavior, and learning effectiveness through their own abilities and efforts (Yufang, 2004). Whether it is good or not only affects students’ self-regulation ability in the process of mathematical learning, as well as their persistence and anxiety in mathematical learning, but also serves as an important mediating variable for learning interest to affect learning effectiveness (Zimmerman, 2000). Currently, research on mathematical self-efficacy mostly focuses on primary and secondary school mathematics education, and there are not many studies on the action mechanism of university mathematical self-efficacy on learning effectiveness.

In mathematical learning activities, students’ learning effectiveness depends to a large extent on whether they can persist in learning, that is, learning persistence, which refers to the degree to which students persist in learning when they encounter difficulties or obstacles. Research shows that learning persistence has an extremely significant impact on learning effectiveness (Jun et al., 2014). Learning persistence is beneficial for students to focus more on the classroom or relevant learning materials. When encountering difficulties, they can continuously avoid distraction through self-regulation and self-management. Research shows that there is a significant positive influence relationship between mathematical learning interest and learning persistence, indicating that mathematical learning interest can affect learning effectiveness through learning persistence (Xiaofeng and Jian, 2017).

Learning anxiety is the anxiety reaction of tension, uneasiness, and even fear that students experience in their minds when participating in classroom learning, answering teachers’ questions, or taking tests or exams. Obviously, it has an important impact on learning effectiveness. Currently, most of the research on learning anxiety is in the primary and secondary school stages. Whether it has an impact on college students in mathematical learning should not be mechanically transferred and needs further verification (Peng and Lihua, 2021). Research shows that one of the main reasons for mathematical learning anxiety is low learning interest (Jun et al., 2015), and at the same time, it has a significant impact on mathematical self-efficacy.

Curriculum-based ideological and political education is an educational concept. It emphasizes the coordinated cooperation between various courses and ideological and political theory courses. Under the “Three-in-One Education” model of involving all staff, throughout the entire process, and in all aspects, it realizes the organic integration of knowledge imparting, ability cultivation, and value guidance and takes “fostering virtue through education” as the fundamental task of education (Deyi and Aidong, 2017). This concept aims to break the situation of separation between knowledge imparting and ideological and political education in traditional teaching, enabling each course to shoulder the mission of ideological and political education. Thus, students can be influenced by correct worldviews, outlooks on life, and values while learning professional knowledge. Mathematics curriculum-based ideological and political education means fully exploring the ideological and political elements in aspects such as mathematicians, the history of mathematics, mathematics culture, the spirit of mathematics, and mathematical thinking, and fully integrating them into curriculum teaching to cultivate students’ awareness and pursuit of truth, goodness, and beauty (Guangfu, 2022).

Compared with other disciplines, curriculum-based ideological and political education has unique and important significance for mathematics education. It is mainly reflected in the characteristics of the mathematics discipline, the cultivation of mathematical thinking, the wide and in-depth application of mathematics, and the cultivation of scientific spirit.

From the characteristics of the mathematics discipline, mathematics is highly logical, abstract, and precise. This makes many students prone to feeling bored and facing difficulties during the learning process, and they may even develop a fear of difficulties and anxiety. Curriculum-based ideological and political education injects humanistic connotations and emotional factors into mathematics education (Mingjuan et al., 2023). For example, by telling the life stories and struggles of mathematicians, such as Zu Chongzhi’s persistent pursuit of the precise calculation of pi under difficult conditions, students can understand that behind mathematical knowledge lies the wisdom and perseverance of countless mathematicians. This can stimulate students’ interest in and motivation for learning mathematics, help them overcome difficulties in the learning process, and enhance their learning perseverance. Due to differences in disciplinary characteristics, this way of combining ideological and political elements with mathematical knowledge may not be as effective in other disciplines as it is in mathematics education in motivating students to overcome the learning obstacles brought about by the discipline itself.

In terms of thinking cultivation, mathematical thinking includes logical thinking, abstract thinking, innovative thinking, etc., and is an important part of students’ comprehensive qualities. Curriculum-based ideological and political education and the cultivation of mathematical thinking complement each other. Incorporating ideological and political elements into mathematics teaching, such as guiding students to view the development of mathematical concepts and theories from a dialectical perspective, can cultivate students’ ability to think comprehensively in connection and development. This not only helps students better understand and master mathematical knowledge but also enables them to transfer this way of thinking to their lives and future work. In contrast, some liberal arts disciplines focus more on the cultivation of imaginal thinking and humanistic emotions. The mathematics discipline plays an irreplaceable role in achieving the integration of thinking cultivation and value shaping with the help of curriculum-based ideological and political education.

Mathematics has extensive and in-depth applications in many fields such as modern science and technology, economics, and finance. It is a fundamental discipline. Through curriculum-based ideological and political education, students can deeply understand the importance of mathematics for national development and social progress and cultivate their sense of social responsibility and mission (Caixia, 2021). For example, when explaining the applications of mathematics in fields such as cryptography and big data analysis, students can be guided to think about how to use mathematical knowledge to contribute to national information security and scientific and technological development. Although other disciplines can also cultivate students’ sense of social responsibility, the mathematics discipline, with its wide range of applications, allows students to understand the social value of their learned knowledge from a more fundamental and universal perspective.

In addition, mathematics curriculum-based ideological and political education helps to cultivate students’ rigorous and realistic scientific spirit (Jie, 2023). The precision of mathematics requires students to be rigorous in dealing with every concept and calculation during the learning and research process, without the slightest carelessness. Under the background of mathematics curriculum-based ideological and political education, the cultivation of this scientific spirit can be internalized into students’ values and codes of conduct through specific teaching cases and ideological and political guidance. Although other disciplines also emphasize the scientific spirit, the mathematics discipline has more advantages in cultivating students’ rigorous scientific spirit due to its unique disciplinary norms.

Therefore, effective mathematics curriculum-based ideological and political education is conducive to cultivating students’ interest in mathematics, helping them overcome the fear of difficulties in mathematics learning, enhancing their self-regulation ability in mathematics learning, and effectively promoting students’ mathematics learning. It is extremely necessary to deeply discuss the impact of mathematics curriculum-based ideological and political education on learning effectiveness, mathematics learning interest, mathematics self-efficacy, mathematics anxiety, and learning persistence.

Learning effectiveness is not merely the accumulation of knowledge. Instead, it is a comprehensive concept that encompasses multi-dimensional gains and improvements of students during the process of mathematics learning. Specifically, it refers to the actual achievements that students have accomplished in aspects such as knowledge acquisition, skill application, thinking development, and learning attitude through a series of mathematics learning activities.

To sum up, mathematical learning interest, as an internal pulling factor for individual students, and ideological and political education in courses, as an external pushing factor for teachers’ teaching, have close internal complex correlations with mathematical self-efficacy, learning anxiety, learning persistence, and learning effectiveness in the process of university mathematics learning. However, previous research often focused on the study of the relationships of a few variables, and most of them used linear regression for analysis. How to reflect the complex relationships of multiple variables and clarify the influence paths among variables has certain theoretical value and practical significance. Based on existing relevant literature, combined with my own teaching practice experience for many years, the author investigates and analyzes data and uses the structural equation model to conduct an empirical study on the influence relationships, degrees, and paths of the six variables, hoping to provide ideas for the reform of university mathematics education and teaching.

## 2 Scale design and research methods

### 2.1 Data source

The research sample was selected from junior and senior engineering students at a university in southwest China. To ensure the randomness and validity of the research data, a series of rigorous processing measures were taken during the survey process.

In terms of the randomization of questionnaire data, professional random sampling software was used to number the 502 questionnaires collected. Random sampling rules were set through the software so that each questionnaire had the same probability of being selected for subsequent analysis. This ensured the randomness and representativeness of the sample and minimized the problem of inaccurate research results caused by sampling bias.

For the elimination of invalid responses, strict criteria and procedures were established. First, a response time threshold was set. The response time of a questionnaire was too short, which is significantly lower than the normal response time required. For example, through pre-testing, it was found that the average time required to complete the questionnaire normally was 20 min. If the response time of a certain questionnaire was <5 min, it could be preliminarily determined that there was a possibility of random filling. At the same time, the consistency of the questionnaire answers was checked. If there were a large number of consecutive identical options (such as more than five consecutive identical options) or the answers had no logical connection with the questions (for example, in questions related to the attitude toward mathematics learning, the answers had nothing to do with mathematics learning), it could be determined as random answering. In addition, questionnaires with extreme answers, that is, all answers were selected as extreme options (such as all choosing “strongly agree” or “strongly disagree”), were also eliminated. According to these criteria, each of the 502 questionnaires was screened one by one. Finally, a total of 26 questionnaires with obvious random and extreme answers were eliminated, and 476 valid questionnaires were obtained, with an effective rate of 94.8%. The situation of the student sample is shown in Table 1.

**Table 1:** Student sample statistics.

Item	Population	Grade		Sex	
		Junior	Senior	Male	Female
Number of students	476	272	204	316	160
Percentage	–	57.1%	42.9%	66.4%	33.6%

### 2.2 Source and Design of the Scale

The student questionnaire drew on the content of academic achievement surveys both at home and abroad. It comprehensively referred to Wu Hongyan’s learning interest scale (Hongyan and Xiaolin, 2017), Bian Yufang’s mathematical self-efficacy scale (Yufang, 2004), Xiong Jianhua’s learning anxiety scale (Jianhua, 2005), and Zhang Lin’s learning persistence scale (Lin and Xiangkui, 2003). Combined with the author’s years of teaching research and practice, a scale of ideological and political education in mathematics courses and learning effectiveness was constructed. It was formed on the basis of two in-depth interviews with 30 students. The research-designed questionnaire adopted the classic 5-point scale form. The items of ideological and political education in courses, learning interest, mathematical self-efficacy, learning anxiety, learning persistence, and learning effectiveness were scored from 1 to 5 points from “strongly disagree” to “strongly agree.” The higher the score, the stronger the cognitive sense of the corresponding variable.

The ideological and political education in courses scale was designed with four items, namely, “Introducing the typical deeds of mathematicians has deeply inspired me,” “Expanding the application of mathematics can promote my active learning of mathematics,” “Introducing mathematical culture makes me more interested in mathematics,” and “Frequently training mathematical thinking is beneficial to improving the effect of mathematics learning.” The learning interest scale was designed with five items, namely, “I’m very interested in mathematics,” “I like mathematics classes very much,” “I like to explore the origin and development of mathematical formulas and theorems,” “I like to read extracurricular reading materials related to mathematics,” “I often feel very happy when talking about mathematics-related issues.” The mathematical self-efficacy was designed with four items, namely, “I think I can learn mathematics well with my own ability,” “I think I’m doing well in mathematics learning,” “I have spent enough time and energy in mathematics learning,” and “I can effectively solve difficulties and interferences when learning mathematics.” The learning anxiety was designed with five items, namely, “I feel anxious when talking about mathematics,” “I feel anxious about the upcoming mathematics class,” “I will feel anxious when submitting mathematics learning assignments or achievements,” “I will feel anxious when answering teachers’ questions,” and “I will feel anxious about the upcoming mathematics exam.” The learning persistence was designed with four items, namely, “I will make an overall learning plan and can stick to learning according to the plan,” “I can stick to not talking to classmates or doing other things in class,” “I can stick to completing homework after class as required,” and “The learning goals I set for myself will not be changed because of other things.” The learning effectiveness was designed with three items, namely, “I can achieve my own goals for mathematics learning,” “My mathematics learning effectiveness can meet the needs of professional learning,” and “Mathematics learning has laid a good foundation for me to review mathematics for postgraduate entrance examination.”

### 2.3 Research hypotheses and model

Based on the previous content and the review of existing research, the author puts forward the following research hypotheses regarding the impact of the promotion of “ideological and political education in university mathematics courses” and the stimulation of students’ “learning interest” on “learning effectiveness.”

*H1:* Curriculum-based ideological and political education has a significant positive impact on the effectiveness of mathematics learning.

From the perspective of the characteristics of the mathematics discipline, the high logic, abstraction, and precision of mathematics tend to make students feel daunted and anxious. However, curriculum-based ideological and political education can inject humanistic connotations and emotional factors into mathematics education. For example, telling the story of Zu Chongzhi’s calculation of pi can stimulate students’ interest and motivation in learning, help them overcome difficulties, enhance their learning persistence, and thus improve their learning effectiveness. In terms of thinking cultivation, integrating ideological and political elements can help students better understand and master mathematical knowledge and transfer mathematical thinking to their lives and work. Due to the wide application of mathematics, through curriculum-based ideological and political education, students can recognize the importance of mathematics for national development and social progress, cultivate a sense of social responsibility and mission, and be more motivated to engage in mathematics learning, thereby improving learning effectiveness. In addition, curriculum-based ideological and political education helps to cultivate students’ rigorous and realistic scientific spirit, which is crucial for mathematics learning and conducive to improving learning effectiveness.

*H2:* Learning interest has a significant positive impact on the effectiveness of mathematics learning.

Learning interest is manifested as learning initiative and is an internal non-intellectual psychological response in students’ learning activities. At the college level, students are more mentally mature. When their learning interest is high, they show higher attention, greater effort, and more active participation in the learning process. Existing research shows that there is a significant relationship between mathematics learning interest and the effectiveness of mathematics learning. A strong learning interest will prompt students to actively explore mathematical knowledge, actively participate in classroom discussions, complete after-class assignments, and engage in extended learning, thus more effectively mastering knowledge and skills and improving learning effectiveness.

*H3:* Curriculum-based ideological and political education has a significant positive impact on mathematical self-efficacy.

Curriculum-based ideological and political education, by telling the stories of mathematicians and introducing mathematical culture, can let students understand the wisdom and perseverance of mathematicians behind mathematical knowledge, enabling students to realize that mathematics learning can be achieved through hard work. For example, when students learn that many mathematicians achieved great success under difficult conditions, they will be inspired and believe that they also have the ability to learn mathematics well, thus enhancing their self-efficacy. At the same time, the sense of social responsibility and mission cultivated by curriculum-based ideological and political education makes students feel that learning mathematics is of great significance for their future development, further enhancing their confidence in learning mathematics well.

*H4:* Curriculum-based ideological and political education has a significant negative impact on learning anxiety.

The difficulty of mathematics learning is likely to cause students to feel learning anxiety. The humanistic connotations injected by curriculum-based ideological and political education can alleviate students’ fear of mathematics. For example, by telling the experiences of mathematicians in overcoming difficulties, students can understand that difficulties can be overcome, reducing their fear of mathematics learning. The positive attitude and correct values cultivated by curriculum-based ideological and political education can help students face the challenges in mathematics learning more calmly and reduce their anxiety levels.

*H5:* Curriculum-based ideological and political education has a significant positive impact on learning persistence.

The content conveyed by curriculum-based ideological and political education, such as the fighting spirit of mathematicians and the importance of mathematics in social development, can stimulate students’ learning motivation and sense of responsibility. After students recognize the value of mathematics learning, they will be more willing to overcome difficulties in the learning process and persevere in learning. For example, when students understand the key role of mathematics in scientific and technological innovation, they will be more determined to study mathematics hard to contribute to related fields in the future.

*H6:* Learning interest has a significant positive impact on mathematical self-efficacy.

When students have a strong interest in mathematics, they will participate more actively in mathematics learning activities. In this process, they continuously make progress and achieve success, and these positive experiences will enhance their confidence in their own mathematical learning ability, thus enhancing their self-efficacy. For example, students who like to explore the origins and developments of mathematical formulas and theorems will believe that they have the ability to learn mathematics well when they solve problems during in-depth exploration.

*H7:* Learning interest has a significant negative impact on learning anxiety.

Students with high learning interest enjoy the process of mathematics learning more and have a positive attitude toward it. They will face mathematics learning tasks more proactively instead of being afraid and avoiding them, so their anxiety level is lower. Some research shows that one of the main reasons for mathematics learning anxiety is low learning interest, which also indicates from the opposite side that learning interest has a negative impact on learning anxiety.

*H8:* Learning interest has a significant positive impact on learning persistence.

Interest is the best teacher. When students are interested in mathematics, they are more willing to invest time and energy in learning and are more patient and persistent in overcoming difficulties in learning, thus showing higher learning persistence. For example, students who like to read extracurricular mathematical reading materials will not give up easily due to small setbacks in learning and will maintain their enthusiasm and perseverance in mathematics learning.

*H9:* Mathematical self-efficacy has a significant positive impact on the effectiveness of mathematics learning.

According to social cognitive theory, self-efficacy affects an individual’s cognition, motivation, and emotion. Students with high mathematical self-efficacy firmly believe that they can learn mathematics well. This positive belief will be transformed into a strong internal driving force, prompting them to actively invest time and energy in exploring mathematical knowledge and be more willing to try different problem-solving ideas when facing difficult problems. Students with high self-efficacy are more inclined to choose effective learning strategies, be good at summarizing and generalizing knowledge, constructing knowledge systems, and seeking diverse learning resources, all of which contribute to improving learning effectiveness.

*H10:* Learning anxiety has a significant negative impact on the effectiveness of mathematics learning.

Learning anxiety can make students feel nervous, restless, and even fearful during the learning process. This negative emotion can distract students’ attention and affect their thinking activity and memory, making it difficult for students to concentrate on learning and understanding knowledge, thus having an adverse impact on learning effectiveness. For example, during an exam, overly anxious students may forget the knowledge points they have mastered and cannot perform at their normal level.

*H11:* Learning persistence has a significant positive impact on the effectiveness of mathematics learning.

The learning effectiveness of students depends to a large extent on whether they can persevere in learning. Learning persistence helps students to be more focused on the classroom or relevant learning materials. When encountering difficulties, they can continuously adjust and manage themselves to avoid distraction. Through continuous learning and practice, students can consolidate and deepen their knowledge, improve their problem-solving ability, develop good learning habits, and form a regular learning rhythm, thereby enhancing their learning effectiveness.

**Figure 1:**
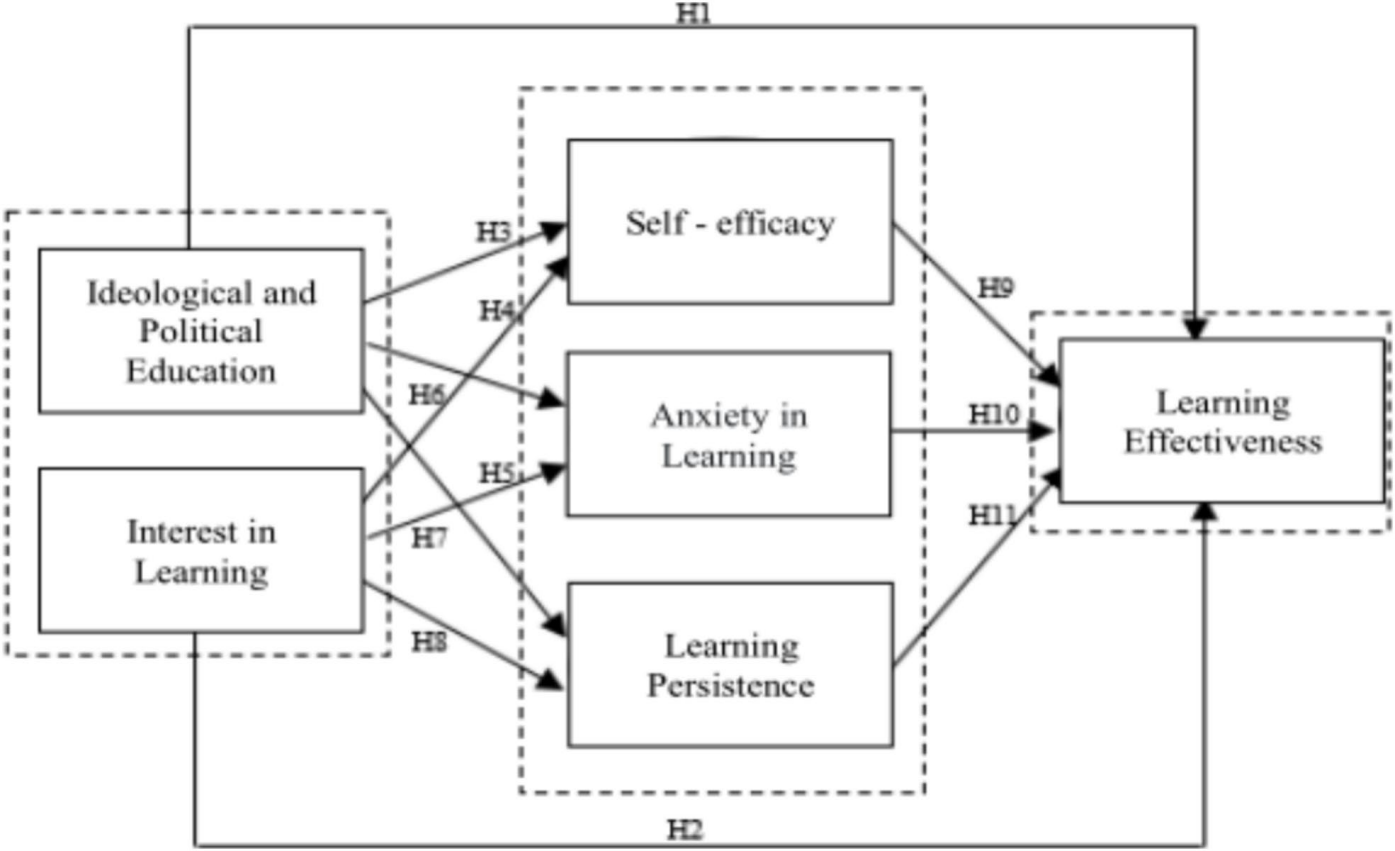
Research model.

Based on the above hypotheses, the research model is constructed as shown in Figure 1. In the research model, ideological and political education in courses and learning interest are independent variables, mathematical self-efficacy, learning anxiety, and learning persistence are mediating variables, and learning effectiveness is a dependent variable.

### 2.4 Data analysis

The research uses SPSS 21.0 and Amos 22.0 software for statistical analysis. The SEM model is mainly used for analysis. First, descriptive statistical analysis and confirmatory factor analysis are carried out for ideological and political education in courses, learning interest, mathematical self-efficacy, learning anxiety, learning persistence, and learning effectiveness to ensure that the variables have a good measurement structure. Then, correlation analysis and multicollinearity test among variables are conducted. Finally, the research model is analyzed to test the goodness of fit of the model and the significance of the relationships among variables.

## 3 Hypothesis testing and result analysis

### 3.1 Reliability testing and factor analysis

Amos 22.0 software is used to conduct exploratory factor analysis on the questionnaire. The results show that the overall alpha value of the questionnaire is 0.912. The composite reliability of each variable, except that the reliability coefficient of “learning persistence” is 0.674, the reliability coefficients of other variables are between 0.779 and 0.928, all >0.7 (Table 2). It is generally believed that the reliability coefficient of a sub-scale between 0.6 and 0.7 is also acceptable (Jichuan et al., 2011). This indicates that the internal consistency of the questionnaire and each sub-scale is good.

**Table 2:** Factor loadings and convergent validity of each variable.

Variable	Question item	Factor loading	Squared multiple correlation	Reliability	Convergent validity
		*Std.*	*SMC*	*Alpha*	*AVE*
Ideological and political education (KSZ)	KSZ1	0.816	0.703	0.886	0.512
	KSZ2	0.783	0.688		
	KSZ3	0.744	0.645		
	KSZ4	0.715	0.622		
Interest in learning (XQ)	XQ1	0.874	0.770	0.928	0.536
	XQ2	0.870	0.768		
	XQ3	0.822	0.716		
	XQ4	0.775	0.672		
	XQ5	0.817	0.708		
Self-efficacy (XNG)	XNG1	0.748	0.646	0.913	0.501
	XNG2	0.684	0.486		
	XNG3	0.735	0.552		
	XNG4	0.729	0.509		
Anxiety in learning (JL)	JL1	0.813	0.749	0.890	0.677
	JL2	0.728	0.643		
	JL3	0.759	0.685		
	JL4	0.864	0.781		
	JL5	0.887	0.792		
Learning persistence (JC)	JC1	0.625	0.462	0.674	0.466
	JC2	0.618	0.401		
	JC3	0.664	0.522		
	JC4	0.611	0.394		
Learning effectiveness (CX)	CX1	0.711	0.595	0.779	0.491
	CX2	0.682	0.481		
	CX3	0.606	0.386		

The distribution of factor loadings of each variable is analyzed. The factor loadings of ideological and political education in courses are between 0.715 and 0.816, the factor loadings of learning interest are between 0.775 and 0.874, the factor loadings of self-efficacy are between 0.684 and 0.748, the factor loadings of learning anxiety are between 0.728 and 0.887, the factor loadings of learning persistence are between 0.611 and 0.664, and the factor loadings of learning achievement are between 0.606 and 0.711, all higher than 0.600, indicating that the measurement variables can better reflect the characteristics of latent variables. The average variance extracted (AVE) of each variable is between 0.466 and 0.677, all close to or >0.500, indicating that the six variables have strong explanatory power for the included items, and the convergence ability of latent variables has reached a relatively ideal state. At the same time, the square multiple correlations (SMCs) of all items are distributed between 0.386 and 0.792, all greater than the medium level of 0.330, and most of them exceed 0.500 and reach the sufficient level of 0.670, indicating that the explanatory power of latent variables for items has reached an ideal situation.

### 3.2 Descriptive statistics and correlation analysis

SPSS 21.0 software was used to conduct descriptive statistical analysis of all variables, and the Pearson correlation coefficient among each variable was calculated and tested. As shown in Table 3, there are significant correlations among the six variables at the *p* = 0.01 level. Among them, there are significant positive correlations between ideological and political education in courses and learning effectiveness, learning interest and learning effectiveness, ideological and political education in courses and self-efficacy, learning interest and self-efficacy, ideological and political education in courses and learning persistence, learning interest and learning persistence, self-efficacy and learning effectiveness, and learning persistence and learning effectiveness. There are significant negative correlations between ideological and political education in courses and learning anxiety, learning interest and learning anxiety, learning anxiety and learning effectiveness, learning communication and self-efficacy, and learning persistence and learning anxiety. Correlation analysis shows that hypotheses H1–H11 can all be accepted. The data results show that the arithmetic square roots of the AVE values of the six variables are all greater than the corresponding correlation coefficients or their absolute values, indicating that there is no obvious multicollinearity among the variables and the discrimination is obvious.

**Table 3:** Descriptive statistics and correlation coefficients of variables.

Variable	KSZ	XQ	XNG	JL	JC	CX
KSZ	**0.716**					
XQ	0.257^**^	**0.732**				
XNG	0.314^**^	0.408^**^	**0.708**			
JL	−0.286^**^	−0.517^**^	−0.394^**^	**0.817**		
JC	0.418^**^	0.545^**^	0.418^**^	−0.302^**^	**0.683**	
CX	0.389^**^	0.492^**^	0.387^**^	−0.463^**^	0.584^**^	**0.701**
Average	3.652	3.322	4.166	3.241	3.103	3.096
Standard deviation	1.060	1.132	0.906	1.154	0.958	1.017

The bold values in the table are the arithmetic square roots of the AVE values. *Indicates a significant correlation at the 0.05 level; **indicates a significant correlation at the 0.01 level.

### 3.3 SEM hypothesis testing

The maximum-likelihood estimation of the research model is conducted, and the model fitting indices are obtained as shown in Table 4. The results indicate that all indices meet the reference standards, suggesting that the SEM model has a good fit and the research model reaches the ideal standard (Jichuan et al., 2011) and can be accepted.

**Table 4:** Test of the fit degree of the SEM model.

Estimation method	*χ^2^*	*χ*^2^/*df*	*RMSEA*	*GFI*	*AGFI*	*CFI*	*TLI*
Maximum-likelihood estimation	857.552	2.334	0.043	0.937	0.916	0.941	0.922
Reference standard	>200	1–3	<0.05	>0.9	>0.9	>0.9	>0.9

A standardized effect analysis of the SEM model is conducted, as shown in Table 4. Ideological and political education in courses (*β* = 0.127, *p* < 0.001), learning interest (*β* = 0.512, *p* < 0.001), self-efficacy (*β* = 0.460, *p* < 0.001), and learning persistence (*β* = 0.495, *p* < 0.001) have a significant positive impact on learning effectiveness; ideological and political education in courses has a significant positive impact on self-efficacy (*β* = 0.154, *p* < 0.001) and learning persistence (*β* = 0.418, *p* < 0.001); learning interest has a significant positive impact on self-efficacy (*β* = 0.336, *p* < 0.001) and learning persistence (*β* = 0.392, *p* < 0.001); ideological and political education in courses (*β* = 0.351, *p* < 0.001) and learning interest (*β* = 0.184, *p* < 0.001) have a significant negative impact on learning anxiety; learning anxiety has a significant negative impact on learning effectiveness (*β* = 0.404, *p* < 0.001). That is to say, all the above hypotheses are established (Table 5).

**Table 5:** Standardized effects of the SEM model.

Path relationship	Standardized direct effects	Standardized indirect effects	Total effect	*p*-value	Hypothesis
CX ← KSZ	0.127	0.113	0.240	0.000^**^	hold
CX ← XQ	0.512	0.069	0.581	0.000^**^	hold
XNG ← KSZ	0.154	–	0.154	0.000^**^	hold
JL ← KSZ	0.351	–	0.351	0.000^**^	hold
JC ← KSZ	0.418	–	0.418	0.000^**^	hold
XNG ← XQ	0.336	–	0.336	0.000^**^	hold
JL ← XQ	0.184	–	0.184	0.000^**^	hold
JC ← XQ	0.392	–	0.392	0.000^**^	hold
CX ← XNG	0.460	–	0.460	0.000^**^	hold
CX ← JL	0.404	–	0.404	0.000^**^	hold
CX ← JC	0.495	–	0.495	0.000^**^	hold

**Indicates a significant correlation at the 0.01 level (two-tailed).

Furthermore, the mediating effects of self-efficacy, learning anxiety, and learning persistence are tested. A Bootstrap asymmetric confidence interval significance test with 2000 sampling times is conducted (as shown in Table 6). From the results, it can be seen that all confidence intervals do not contain 0, indicating that the mediating effects and their corresponding paths are significant. Since all the standardized direct effects in the research model are significant, the mediating effects in all mediating paths are partial mediation.

**Table 6:** Mediation effect analysis.

Path relationship	Bias-corrected confidence interval (95%)	Significance	Mediation effect
CX ← XNG ← KSZ	[0.156, 0.432]	Significant	Partial mediation
CX ← JL ← KSZ	[0.127, 0.384]	Significant	Partial mediation
CX ← JC ← KSZ	[0.212, 0.425]	Significant	Partial mediation
CX ← XNG ← XQ	[0.096, 0.224]	Significant	Partial mediation
CX ← JL ← XQ	[0.058, 0.197]	Significant	Partial mediation
CX ← JC ← XQ	[0.147, 0.408]	Significant	Partial mediation

If the confidence interval does not include 0, it is significant; otherwise, it is not significant.

## 4 Conclusion and discussion

The research shows that mathematical learning interest and ideological and political education in mathematics courses have a direct and significant positive impact on the effectiveness of mathematical learning, stimulating students’ internal interest, fully exploring students’ psychological perception of mathematics, and forming students’ internal drive for active learning. Pulling students’ learning behaviors through interest is conducive to the improvement of learning effectiveness. Research shows that the internal motivation of college students’ learning is to transform learning interest into learning motivation and improve the effectiveness and sustainability of learning participation (Qian et al., 2018). In teaching practice, as for how to arouse college students’ interest in mathematics learning, teachers, as the leaders in the classroom, should not be limited to simply imparting knowledge or even reading textbooks mechanically. They should fully design various teaching forms and adopt various teaching methods, such as case introduction, classroom discussion, knowledge expansion, and mathematical application. The correlation coefficient between learning interest and ideological and political education in courses is significant at the 0.01 level, indicating that scientific implementation of ideological and political education in courses has a great promoting effect on the improvement of college students’ interest in mathematics learning. Ideological and political education in courses, as an extremely important channel for “three-wide education” and “fostering virtue through education” in mathematics courses, still has a positive pulling effect on the effectiveness of mathematics learning. Good ideological and political education in courses will ultimately be beneficial to the improvement of learning effectiveness. In the context of the increasingly fierce competition in higher education in the new era, as a very important public basic course, university mathematics subject groups or teaching and research offices should abandon the traditional concept of relying on only one textbook. They should organize the construction of ideological and political education in university mathematics courses, invite professional teachers and ideological and political teachers to participate in teaching and research activities, fully excavate ideological and political elements, design teaching cases of ideological and political education in courses, and promote the diversified and high-level teaching process of university mathematics.

Mathematical self-efficacy, learning persistence, and learning anxiety all show significant mediating effects in the influence of learning interest and ideological and political education in courses on learning effectiveness. The stronger students’ interest in mathematics is, the more it will drive students’ learning motivation and degree of effort, and promote students to form a positive, forward-looking, and controllable self-efficacy. Research shows that learning motivation based on self-efficacy can promote students to actively control learning goals, effort levels, and learning persistence in difficult situations, thus ensuring the achievement of goals (Bandura, 1989). According to the self-regulated learning theory, when individuals face learning difficulties, students who can persevere will devote more emotions, make more efforts, and can effectively regulate and control their own learning state, thereby ensuring the quality and efficiency of learning (Jun et al., 2014). Learning anxiety is significantly negatively affected by learning interest and has a significant negative impact on learning effectiveness. Mediation effect analysis shows that mathematical learning anxiety has the function of mediating learning interest and learning effectiveness, which is consistent with existing research results (Gilman and Anderman, 2006). It indicates that gradually cultivating students’ interest in mathematics can effectively reduce students’ anxiety and fear of mathematics, thereby improving learning effectiveness.

Self-efficacy, as a dispositional variable, reflects an individual’s subjective judgment and confidence in their own mathematical learning ability. Learning persistence, as a behavioral variable, embodies an individual’s actual performance of continuous learning in the face of difficulties during mathematics learning. Both have a significant impact on the effectiveness of mathematics learning. In terms of self-efficacy, students with high self-efficacy firmly believe that they can learn mathematics well. This positive belief is transformed into a strong internal motivation. When faced with complex formula derivations and difficult problem-solving, they are more willing to actively invest time and energy to explore solutions. In contrast, students with low self-efficacy tend to doubt their abilities and lack the willingness to learn actively. Students with high self-efficacy are also more inclined to choose effective learning strategies. They are good at summarizing and inducting knowledge, constructing knowledge systems, and actively seeking diverse learning resources. However, students with low self-efficacy are confined to traditional methods due to a lack of confidence. When facing difficulties, students with high self-efficacy regard them as opportunities to improve their abilities and respond actively, while students with low self-efficacy are prone to anxiety and a tendency to avoid. Regarding learning persistence, students with high learning persistence can ensure sufficient investment of learning time and energy. They consolidate and deepen their knowledge through repeated practice and in-depth thinking about knowledge points. For example, by doing exercises repeatedly, they can master the solution methods of various question types proficiently. They can also develop good learning habits, form a regular learning rhythm, and improve learning efficiency. Moreover, when facing fluctuations in learning performance, they will not give up due to setbacks. Instead, they can maintain the stability of learning, adjust their methods, and continue to make progress. In contrast, students lacking learning persistence may interrupt their learning or change their plans when their academic performance is unsatisfactory. In addition, self-efficacy and learning persistence influence each other. High self-efficacy enhances learning persistence, and the improvement of learning persistence further strengthens self-efficacy. The two promote each other and jointly contribute to the improvement of the effectiveness of mathematics learning.

Appropriate ideological and political education in courses is not only conducive to cultivating students’ correct world outlook and outlook on life but also conducive to shaping students’ strong character, positive and optimistic attitude toward handling affairs, and effective self-regulation ability when encountering difficulties, which is beneficial to students’ formation of good self-efficacy. The research results show that ideological and political education in courses has a significant positive impact on mathematical self-efficacy and learning persistence and a significant negative impact on learning anxiety, indicating that in the path of ideological and political education in courses—learning effectiveness, self - efficacy, learning persistence, and learning anxiety have obvious mediating effects.

From the perspective of the overall effect of model analysis, the impact on the effectiveness of mathematical learning is as follows: mathematical learning interest (0.581) > learning persistence (0.495) > self-efficacy (0.460) > learning anxiety (0.404) > ideological and political education in courses (0.240); the comprehensive impact of learning interest is as follows: mathematical learning effectiveness (0.581) > learning persistence (0.392) > self-efficacy (0.336) > learning anxiety (0.184); the comprehensive impact of ideological and political education in courses is as follows: mathematical learning anxiety (0.418) > learning persistence (0.351) > learning effectiveness (0.240) > self-efficacy (0.154). Therefore, in university mathematical teaching activities, we should not only attach importance to cultivating students’ will, spirit, and quality through effective ideological and political education in courses to promote students’ persistent learning but also consciously cultivate students’ interest in mathematics, stimulate students’ internal driving force for learning, and promote students’ active learning. Through internal-cause pulling and external-cause pushing, it can not only effectively relieve learning anxiety but also promote students’ persistent learning and improve mathematical self-efficacy and learning effectiveness.

## 5 Research significance and research prospects

The research has analyzed the influence mechanism of ideological and political education in university mathematics courses and learning interest on learning effectiveness and conducted an empirical analysis of the influence path and degree. The results have certain theoretical significance and practical value. The research results show that mathematical learning interest, ideological and political education in courses, self-efficacy, learning persistence, and learning anxiety all have important impacts on learning effectiveness. For current university mathematics education, especially in the overall context of “three-wide education,” teachers’ educational and teaching activities should not be limited to completing teaching tasks. They should pay attention to the goal—orientation, vividness, and challenge of the classroom, effectively carry out ideological and political education in courses, cultivate students’ learning interest, stimulate students’ initiative, enthusiasm, persistence, and self-regulation and monitoring awareness in learning, let students highlight their subjective initiative, actively participate in mathematical activities, and form an organic closed-loop mechanism for improving the effectiveness of mathematical learning.

The research still has certain limitations. First, the reliability of the questionnaire data completely depends on students’ truthful responses. When formulating the learning effectiveness scale, only three self-designed items were considered, and the intention of taking the postgraduate entrance examination was investigated, without using students’ test scores. Since there are some students who have no plans to take the postgraduate entrance examination, this has affected the universality of the dependent variable to a certain extent. Further optimizing the questionnaire variable items and expanding the survey subjects are issues that need to be focused on in the next step of the research. Second, there are many factors affecting learning effectiveness, and their relationships are rather complex. Variables such as mathematical cognition and learning motivation were not selected in this study. The impact mechanisms and paths of these factors on learning effectiveness in contemporary college mathematics education remain to be further analyzed in depth. Third, in this research, only the mediating effects of self-efficacy, learning anxiety, and learning persistence were analyzed. Their moderating effects, the existence of other moderating variables, and the mutual influence relationships among them have not been clearly and further analyzed. This is also research that needs to be carried out in the next step.

## Data Availability

The original contributions presented in the study are included in the article/supplementary material, further inquiries can be directed to the corresponding author.
